# Effect of *Ceratonia siliqua* (Carob) syrup and vitamin E on sperm parameters, oxidative stress index and sex hormones in infertile men: Protocol for a randomized controlled trial

**DOI:** 10.22088/cjim.10.4.452

**Published:** 2019

**Authors:** Mir Mohammad Reza Aghajani, Neda Mahdinezhad Gorji, Parvaneh Mirabi, Faraz Mojab

**Affiliations:** 1 Infertility and Reproductive Health Research Center, Health Research Institute, Babol University of Medical Sciences, Babol, Iran; 2 *Pharmaceutical Sciences Research Center and School of Pharmacy, Shahid Beheshti University of Medical Sciences, Tehran, Iran*

**Keywords:** Male infertility, Ceratonia siliqua, Sperm parameters, Oxidative stress, Sex hormones

## Abstract

**Background:**

Spermatogenesis is a necessary process in fertility and reproductive capacity of humans. In view of the relative high prevalence of spermatogenesis disorders in men and the failure of treatments provided in conventional medicine, new therapeutic approaches are being considered. This study will be designed to investigate the effect of Ceratonia siliqua (Carob) syrup and vitamin E on sperm parameters, oxidative stress index and reproductive hormones in infertile men.

**Methods:**

This randomized controlled trial protocol will be set in Babol Uuniversity of Medical Sciences. The proposed sample size is 60 men with oligozoospermia and teratospermia with 30 men in the experimental group taking Carob syrup while 30 men in the comparison group receiving vitamin E. The primary outcome measure is the change in semen parameters and secondary outcome measures including change in endocrine parameters and stress oxidative markers. This research was registered in the website of Iranian Registry of Clinical Trials as IRCT20171209037794N1identification number

**Conclusion:**

If the beneficial effect of this herb can be confirmed, it will provide a cost-effective method for helping infertile men and provide evidence-based references for the treatment of male infertility in future.

R Disorders of production of sperm during spermatogenesis process is one of the most common causes of male infertility. In view of the relative high prevalence of spermatogenesis disorders and the failure of treatments provided in conventional medicine, new therapeutic approaches are being considered (1). In recent years, the increasing use of medicinal herbs has led to extensive research on herbal medicine. Many sperm changes in idiopathic infertility are associated with high levels of reactive oxygen species (ROS). Increased production of ROS leads to oxidative damage to lipids, proteins, and cellular DNA. The damage to sperm’s DNA results in reduced sperm motility, acrosome membrane damage, and sperm disability in fertilizing oocyte (2, 3) so herbs with an antioxidant capacity can cope with the destructive effects of free radicals produced and the oxidative stress inside minimize the cell in infertile male. On the other hand, inflammation also reduces antioxidant’s defensive power (3). Various studies have reported that supplementation with antioxidants reduces inflammatory factors (4-7). One of these medicinal plants is *Ceratonia siliqua* (Carob) tree from Legominasae family which has a height of 7-12 meters.

It is indigenous in the Mediterranean region, southern Syria, and India, as well as California, and grows widely in Iran, especially in Shapur, Fars and Kazeroun. This herb contains a lot of fibers, carbohydrates, polyphenolic compounds, lignin, protein, calcium, potassium phosphorus, glutamic acid, linolenic acid, Linoleic acid, vitamins E, D, C, B6, folic acid, iron, and magnesium. It is also a natural source of antioxidants of which its anti-oxidant activity is related to tannin and phenol compounds (8-11).

It is traditionally used to increase sperm count (8, 12). Animal studies showed that using the extract of this herb have influences on cAMP production and activity of enzymes involved in rat's testicular steroidogenesis. In these researches, the use of Carob extract improved sperm quality, increased testosterone levels and biochemical parameters by affecting leydig cells and biosynthesis of testosterone, so is effective on sex hormones, sperm production, and spermatogenesis process (8, 11). The major mechanisms behind these effects are unknown. There have been no controlled human clinical trials to evaluate the effects of *Ceratonia silique* on spermatogenesis in infertile men. However, it is used locally in many countries**.** No side effects have been reported for the use of this herb (fruit, capsule, or extract) .Researchers have reported no particular complications and have declared that its consumption is permitted in humans (12, 13). Based on our knowledge, human studies have not investigated the effects of this herbal extract on sperm parameters, oxidative stress indices, and the level of reproductive hormones or the results have not yet been published. Given the fact that there are still problems in the treatment of male infertility in new medicine, and given that in recent years, herbal medicine has been widely used as complementary medicine, the present study was designed to investigate the effect of *Ceratonia siliqua* syrup and vitamin E on sperm parameters, oxidative stress index and reproductive hormones in infertile men. 

## Methods

**Study design: **The protocol of this randomized controlled trial study has been approved by the Institutional Review Board of Babol University of Medical Sciences (IR.MUBABOL.HRI.REC.1396.179). 

**Study setting: **This study will be conducted at the Infertility and Reproductive Health Center affiliated to Babol University of Medical Sciences and at the urology clinic. 

**Sample size: **The trial is powered to detect an effect size of d ≥0.70 as statistically significant in a two-tailed test with α=0.05 and power of 0.80 with N=26 per condition. As there is the possibility that some patients do not complete the study, we will include 30 patients in Carob and vitamin E groups.

**Participants: **Male partners of couples attending the infertility center of Babol University of Medical Sciences will be included in the study. The proposed sample size is 60 men with teratospermia and oligozoospermia with 30 men in the experimental group taking Carob syrup and 30 men in the comparison group and receiving vitamin E. 

**Eligibility criteria: **The eligibility of each included subject will be confirmed by the urologist. Patients eligible for the trial must comply with all of the following at randomization:

- 20-45 years of age, who had complained of infertility for at least 12 months, and had no history of surgical or medical treatments for infertility.
- Having a BMI less than 30.
- Uncertain oligo-spermatozoa due to WHO criteria (sperm concentration less than 15 million per ml, type A motility less than 25%, and type B mobility Less than 50%, normal morphology less than 15%).
- Not smoking, drinking alcohol, and addiction to substance.
- Lack of systemic illness diseases, such as diabetes and thyroid disease, cerebrovascular disease and had no history of chemotherapy.
- Naturally occurring gonadotropins, testosterone and prolactin

- Lack of history of vasectomy or obstructive azoospermia.
- Patients with recurrent or residual varicocele, and history of cryptorchidism or Azoospermia and chromosomal abnormalities such as kleinfelter's Syndrome are not enrolled in the study.

**Exclusion Criteria:**

- Genital tract infection.
- The use of antioxidant supplements in the last three months and during the study.
- The use of chemical drugs during the study.
- Having severe physical activity.
- Hypogonadism or pituitary abnormalities.

**Recruitment: **In the run-in phase of the study, a member of the research team (reproductive health researcher) and urologist present at Fatemezahra Infertility Clinic 3 days in a week. The investigators will inform the participants of all aspects pertaining to participation in the study and will screen all the patients for the eligibility criteria at the time of admission. Subjects` screening will be continued until the target population is achieved. Participants will be free to withdraw any time during the study, and this will not affect their clinical treatment.

**Pre-randomization data: **At the initial visit, baseline clinical demographics and a complete medical history will be obtained through a clinical interview, including age, occupation, body mass index, cigarette use, alcohol intake, medical history, drug history and a working clinical diagnosis. A physical examination will be performed in all who have a disorder in the number, motility or morphology of the sperm to identify any potentially eligible infertile men for the study. The criteria for choosing patients with disorders in terms of sperm count (oligospermia) abnormal morphology (teratospermia) and reduced sperm motility (asthenospermia) as well as the statistical results of the semen analysis are based on the WHO criteria (14). After the urologist determines that the patient is eligible for recruitment, all eligible participants will be asked to sign a written consent form before being enrolled in the study.

**Blinding: **The researchers performing and analyzing data from the semen samples, endocrine tests, and biomarker analysis (outcomes assessor) will be blinded to the participant grouping. 

**Allocation (method & allocation concealment mechanism): **The block randomization method will be used to randomize patients into groups that result in equal sample sizes to ensure a balance across groups over time.

Before initiation of the run- in phase of the study, two 60- sets of random numbers will be created by a member of the research team not involved in recruitment. 15 blocks with size of 4 and combination of A for the carob and B for vitamin E group will be prepared.

Allocation sequence will be password-protected and only accessible to the reproductive health researcher. She folds two times the paper containing 4-size blocks, put them in the standard thick envelopes and writes the serial numbers on them. All of the envelope will be kept at the recruitment center. Thus, for every eligible patient, the researcher randomly selects one of the envelopes after shuffling and assign subjects into intervention and comparison group.

**Data collection: **After physical examination and check baseline characteristic data and eligibility criteria, semen analysis and hormonal status, including serum follicle-stimulating hormone (FSH), prolactin, luteinizing hormone (LH), total testosterone and total antioxidant capacity (TAC) and malondialdehyde (MDA) will be done at baseline and 3 months after enrollment.

The semen samples will be collected in the privacy of a room near the laboratory after three days of sexual abstinence and kept at 37°C. A validated and standard laboratory (Fatemezahra Infertility Center) will be used for collection and analyses of the semen. Sperm characteristics will be studied on fresh samples immediately after collection. After liquefaction seminal fluid at room temperature, routine semen analyses including semen volume, concentration, sperm motility and morphology will be performed according to WHO recommendation (14). 

Blood samples will be centrifuged and serum will be stored at -50°C for metabolomics biomarkers that include oxidative stress index and sex hormones. Measuring the sex hormones is done by ELISA method and according to instruction of the related kit.

**Participant timeline: **The time schedule of enrolment, interventions, and assessments based on SPIRIT 2013 template (15) has been shown in Table 1.

**Table 1:** SPIRIT 2013 schedule of enrolment, interventions, and assessments

	** Study period**					
	**Enrolment**	**Allocation**	**Post allocation**			**Close- out**
**Time point**	**-t**_1_	**0**	**t**_1_	**t**_2_	**t**_3_	**t**_x_
**Enrolment**	X					
Eligibility screen	X					
Informed consent	X					
Medical history	X					
Safety index	X					
FSH, LH, Prolactin, MDA Testosterone, TAC level	X					
Semen analyses Allocation	X					
		X				
**Intervention**						
Carob group		↔				
Vitamin E group			X	X	X	
**Assessments:**						
FSH,LH,Prolactin, MDA Testosterone, TAC level						X
Semen analyses						X
**Adverse events**			X	X	X	X

**Data Monitoring: **Two researchers from BUMS not involved in the study and chosen by the university will monitor the data and supervise the conduct of the study.

**Figure 1:**
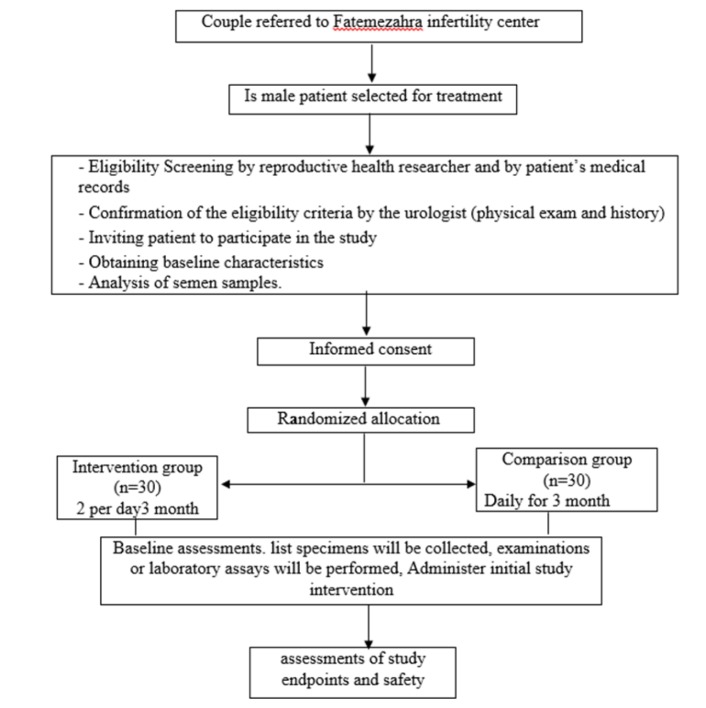
Flowchart of the study

**Intervention: **Patients will be assigned (1:1) to receive Carob syrup twice a day or vitamin E (comparison group) 100 mg once a day for 3 months. The Carob pods will be purchased from herbal market in Tehran and after identification and confirmation by botanist will be powdered and extracted by decoction in water (100 gram Carob, (traditional dosage) in 500 ml water). Then, after filtration, will be mixed with 500 ml USP syrup. Each patient will use 15 ml of Carob syrup/day (divided ×2) (~ dosage in traditional medicine). For all patients, a spermogram is performed first. Three months after the treatment, the spermogram and blood samples were taken from the two groups. The main consequence of the study is to improve the spermogram test and the secondary outcome of each of the parameters of prolactin, FSH, TAC, MDA and testosterone. During the initial visit where recruitment of the study occurs, the participants will be prescribed the medication. It is initially prescribed for two weeks. One week later, a telephone interview will be conducted to review the side effects and arrange an early appointment with the patient’s request. Two weeks later, the patient will received the remaining medications and adherence to treatment will be assessed (Figure 1).

**Follow-up: **Participants will be monitored monthly for signs or symptoms of adverse effects. One month later, the participants will be reviewed in the clinic and a possible side effect check list will be conducted. To prevent attrition and to assess adherence to treatment, telephone interviews will be conducted again at 2 months from administration of the drug. 3 months after intervention and upon completion of the drugs, all participants will be checked in the infertility clinic, semen analysis will be performed according to the standard clinic protocols for the sperm count, motility and morphology and serum samples will also be collected in order to evaluate markers of oxidative stress and reproductive hormones (Figure 1).

**Adherence: **Self-assessment patient’s checklist will be used to assess adherence to treatment, also participants will instruct to bring their Carob syrup bottle each visit.

**Outcome measures: **The primary outcome measure will be the change in semen parameters after 3 months in men who were receiving Carob syrup compared to men who were taking vitamin E. Primary outcome measure according to (WHO) methodologies.

 [Time frame: Before treatment and after 3 months treatment were finished in 2 treatment arms, semen analysis will be performed].

1. sperm concentration, 2) percent motility, 3) sperm morphology Secondary outcome measures will include the change in endocrine parameters, stress oxidative markers and assessment of the adherence to treatment.

1. Changes in serum levels of reproductive hormones after treatment ( Time frame: Before treatment and after 3 month treatments were finished in 2 treatment arms, reproductive hormones will be performed) Serum concentrations of follicle-stimulating hormone, luteinizing hormone, total testosterone and prolactin.
2. Evaluate the level change of oxidative stress biomarkers in Serum (Time frame: Before treatment and after 3 months treatment were finished in 2 treatment arms, oxidative stress biomarkers will be performed) Serum concentrations of Malondialdehyde (MDH) and total antioxidant capacity (TAC) using spectrophotometer at 532 nm.
3. Self-assessment patient’s checklist will be used to assess adherence to treatment. Reasons for dropping out and non-compliance will also be noted and reported.

**Statistics and data-analysis: **Baseline characteristics will be compared between Carob and vitamin E groups using chi-squared tests for categorical variables and independent *t*-test for the normally distributed continuous variables. If the assumptions for normality are violated, then non-parametric techniques will be applied. One-way analysis of covariance (ANCOVA) will be used to evaluate treatment effects of Carob. Relative associations among parameters will be determined by Pearson’s correlation coefficient. All statistical analyses will be conducted using SPSS (Version 16) software. A p-value of less than 0.05 (two-sided) indicates a statistically significant difference, with 95% confidence intervals.

## Discussion

Over the past decade, diagnosis of male reproductive

performance has progressed dramatically. Many infertile men have disorders treatable with medication that can be eliminated, if properly diagnosed and treated, which will allow natural fertilization. Currently, 60% of the world’s population use herbal drugs for treatment of diseases and 25% of physician’s prescriptions include herbal compounds that have less adverse effects than other drugs. Infertility is one of the medical issues of couples and according to the World Health Organization (WHO), 10% of couples are affected with this problem, of which 10% to 40-50% are related to male factor, the most important is the low quality of sperm. Therefore, paying attention to eliminating these disorders is very important for treatment of couples ‘infertility. There are numerous medicinal herbs with anti-fertility and fertilizing effects in the world. These herbs have been used throughout the history to decrease or increase fertility in men. Many people now use herbal medicines or their derivatives to increase fertility and sexual desire. Thus, it is necessary to study the herbal biologically active substance on male fertility and natural herbal substances with estrogenic and anti-estrogenic properties (5). The WHO has reported that despite the increasing use of herbal medicines, there is still significantly insufficient research information, while the role of articles that examine the herbs is very important. Considering the clarification of adverse effects of chemical drugs on human, women and men have increasingly tended toward using herbal drugs. Herbal drugs are an appropriate substitute for normal drugs.

This is the first study to compare the changes in sperm parameters after two different interventions: the active intervention (Carob) and vitamin E intervention. If successful, this study will provide new knowledge on preferred procedures for helping men and couples to conceive and offer guidance to urologists treating these men.

***List of abbreviations:***

Reactive oxygen species (ROS)

*Ceratonia Siliqua* (Carob)

Cyclic AMP (CAMP)

Body mass index (BMI)

Follicle-stimulating hormone (FSH)

Luteinizing hormone (LH), 

Total antioxidant capacity (TAC) 

 Malondialdehyde (MDA)

*Declarations*

**Ethics approval and consent to participate:** The research project has received the confirmation of the Institution Ethics Committee (Babol University of Medical Sciences) with the number 5121 dated 3/2/2015. A written consent will be obtained from each patient for the use of blood samples.

**Consent for publication:** The consent form of our patients will be taken and will be available.

**Availability of data and materials:** The datasets during the current study available from the corresponding author on reasonable request.
